# Interleukin-17 receptor C gene polymorphism reduces treatment effect and promotes poor prognosis of ischemic stroke

**DOI:** 10.1042/BSR20190435

**Published:** 2019-10-11

**Authors:** Jing Tian, Yongjie Bai, Aimin You, Ruile Shen, Junqiang Yan, Wenjing Deng, Lijun Wen, Meng Li, Junfang Teng

**Affiliations:** 1 Department of Neurology, The First Affiliated Hospital of Zhengzhou University, Zhengzhou 450052, Henan Province, China; 2 Department of Neurology, First Affiliated Hospital, and College of Clinical Medicine of Henan University of Science and Technology, Luoyang 471003, China; 3 Department of Rehabilitation Medicine, First Affiliated Hospital, and College of Clinical Medicine of Henan University of Science and Technology, Luoyang 471003, China; 4 Department of Neurological Intensive Care Unit, The First Affiliated Hospital of Zhengzhou University, Zhengzhou 450052, Henan Province, China

**Keywords:** IL-17RC, Ischemic stroke, Prognosis, Re37511

## Abstract

**Objective:** To study the relationship between Interleukin-17 receptor C (IL-17RC) gene polymorphism and ischemic stroke (IS).

**Methods**: Three hundred cases of IS patients and 300 cases of the healthy controls were selected. Serum of IS patients and the controls was collected. The relative mRNA levels of IL-17, IL-17RC, IL-6, IL-8, G-CSF and granulocyte-macrophage colony stimulating factor (GM-CSF) by qRT-PCR. The protein expression of IL-17 and IL-17RC was determined by Western blotting. IL-17RC genotype was identified by PCR amplification. The proportion of IL-17RC, SNP and re37511 in IS and control group was determined. The treatment effect on IS and prognosis of patients with IL-17RC, SNP and re37511 was compared.

**Results:** The relative mRNA levels of IL-17, IL-17RC, IL-6, IL-8, G-CSF and GM-CSF in IS group were significantly higher than the control group. The protein expression of IL-17 and IL-17RC in IS group was also markedly higher than the control group. The proportion of IL-17RC re37511 in IS group was much larger than control group and proportion of IL-17RC much less. The percent of poor treatment effect in re37511 was much larger than IL-17RC. The percent of death and recrudescence in patients with IL-17RC re37511 was the highest.

**Conclusion**: IS up-regulates the expression of IL-17 and IL-17RC. IL-17RC re37511 indicates the patients have a poorer treatment effect and prognosis.

## Introduction

Stroke is the most common reason of permanent disability in adults in the world [1]. After stroke for 3 months, 20% of the survivors need nurse and 15–30% become permanently disabled [2]. Pathogenesis of stroke includes a variety of mechanisms, but inflammation and ischemic damage are the major reasons. Ischemic stroke (IS) is a serious disease and usually associated with a high rate of mortality [3]. Atherosclerosis is one of the main causes of IS and its important pathophysiological basis is inflammation of blood vessels. Therefore, the levels of pro-inflammatory genes can affect the occurrence and development of IS.

IL-17, secreted by CD4^+^ activated memory T cells, is a homodimeric polypeptide and its molecular weight is approximately 20–30 kDa. IL-17 cytokine family includes IL-17A, IL-17B, IL-17C, IL-17D, IL-17E and IL-17F. The receptor family of IL-7 contains five members: IL-17RA (receptor A), IL-17RB, IL-17RC (receptor C), IL-17RD and IL-17RE. Functionally, IL-17 is a pro-inflammatory regulatory factor and can lead to inflammation in leptocytes. It can play a physiological role only after binding to the receptor, while interleukin-17 receptor (IL-17R) can be expressed on the surface of vascular endothelial cells. In endothelial cells, IL-17 combines with IL-17R to activate NF-κβ and proteases P-38, which then accelerate the secretion of IL-6, IL-8 and prostaglandin E2 (PGE2). Some studies showed IL-17 alone or combination of IL-17 and TNF-α can result in the synthesis of IL-6 in fibroblast-like synovial cells [4]. IL-17C often regulates the activities of epithelial cells via autocrine role, which increase the levels of antimicrobial peptides and inflammatory cytokines in epithelial cells [5]. IL-17C regulated signaling pathway has a key role in innate mucosal immune response. In epithelial cells, IL-17C and *Pseudomonas aeruginosa* collaboratively increase IL-6 expression [6]. Many studies also showed release of mucin, IL-6, IL-8 and granulocyte-macrophage colony stimulating factor (GM-CSF) after stimulation on epithelial cells. Those factors (IL-6, IL-8 and GM-CSF) can enhance the accumulation of neutrophils [7].

In the present study, we aimed to explore the relationship between IL-17RC gene polymorphism and development of IS.

## Materials and methods

### Study population

Between April 2015 and May 2016, 300 patients, who were recently diagnosed with IS, were recruited from Qilu Hospital of Shandong University Hospital. Moreover, 300 healthy people were taken as control group. Both IS patients and the controls, gender and age-matched (±5 years) were selected. Before our study, all participants signed written informed consents. Our study protocol was also approved via ethics committee of Qilu Hospital of Shandong University Hospital. The serum of IS patients and controls was collected.

### Quantitative real time PCR (qRT-PCR)

Total RNA were isolated from tissue or cells with TRIzol kit. RT-PCR was performed in a Mastercycler RT-PCR detection system (Eppendorf, Westbury, NY). Relative expression level of genes was normalized against gene β-actin by formula of 2^Δ*C*^_T_^ (β-actin-target)^. Gene level in experimental group was then normalized against the control group.

### Western blotting

Western blot analysis was performed as previously described. Antibodies for IL-17 and IL-17RC were from Abcam and antibody for β-actin was from Sigma. The band intensities were quantified by Storm 860 Molecular Imager (GE).

### Genotype analysis

Venous blood (5.0 ml) was obtained from each participant and stored at −20°C for use. Genomic DNA was extracted from the blood by TIANamp blood DNA kit (Tiangen Biotech Co., Ltd, Beijing, China). PCR-restriction fragment length polymorphism (RFLP) analysis was applied to detect the genotyping of SNPs rs37511 in IL-17RC gene. Primers were designed by MassARRAY® Assay Design software version 4.0 (Sequenom, San Diego, CA, U.S.A.) and are shown in Table 1. The cycle procedure was as follows: 95°C for 5 min, 35 cycles at 95°C for 30 s, 62°C for 30 s, 72°C for 30 s and finally 72°C for 10 min. PCR products were stained by ultraviolet light and Ethidium Bromide, and then sequenced by automated sequencing system.

**Table 1:** Primer for SNPs rs37511

5′→3′	Forward	Reverse
	TCCTTAGCAACCATTAATCTGG	AAATAGCAAAAACTGACACAAGGC

### Statistical analysis

SPSS 22.0 software was used to perform *t* test and variance analysis. Significance was defined as *P*<0.05.

## Results

### IS up-regulates IL-17, IL-17RC, IL-6, IL-8, G-CSF and GM-CSF

**Figure 1:**
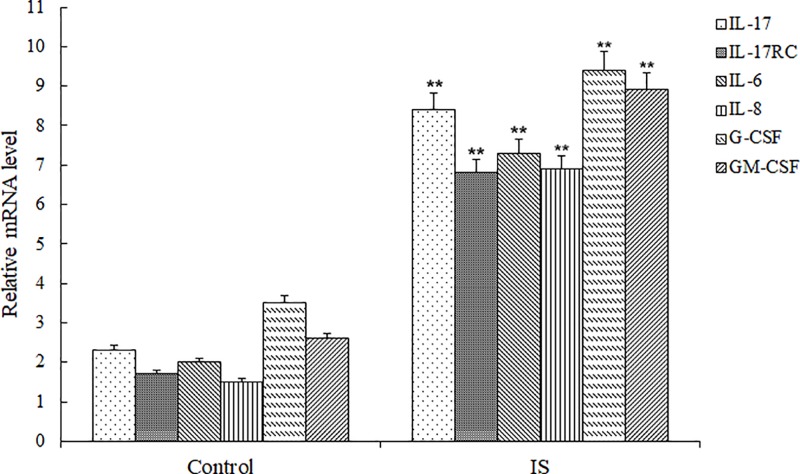
The relative mRNA levels of IL-17, IL-17RC, IL-6, IL-8, G-CSF and GM-CSF in serum samples from IS by RT-PCR Compared with the Control group, **P <0.01.

**Figure 2:**
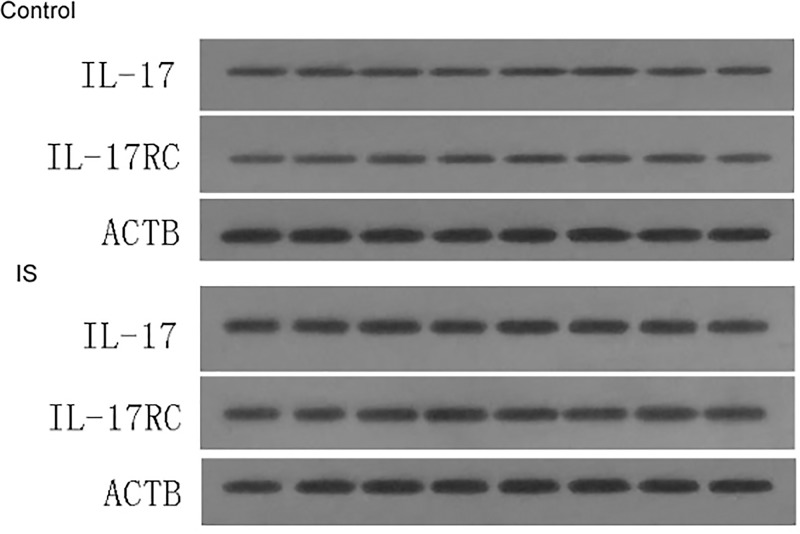
The protein expression of IL-17 and IL-17RC in serum samples from IS by Western blotting

To explore the relationship between inflammation and IS, we determined the mRNA levels of IL-17, IL-17RC, IL-6, IL-8, G-CSF and GM-CSF by qRT-PCR. The results of RT-PCR are shown in Figure 1. As shown, the relative mRNA level of IL-17 in IS group was 8.91 ± 0.75, which was significantly higher than that in the control group of 2.34 ± 0.51 (*P*<0.01). Similarly, the relative mRNA levels of IL-17RC, IL-6, IL-8, G-CSF and GM-CSF in IS group were all markedly higher than those in the control group. Western blotting was used to verify the expression change of IL-17 and IL-17RC in IS and results are shown in Figure 2. As shown, the protein expression of IL-17 and IL-17RC in IS group were stably higher than the control group (*P*<0.01). Therefore, we speculated IS up-regulated the expression of IL-17, IL-17RC, IL-6, IL-8, G-CSF and GM-CSF, and the expression of IL-6, IL-8, G-CSF and GM-CSF were induced by IL-17, IL-17RC.

### Identification of IL-17RC genotype

**Figure 3:**
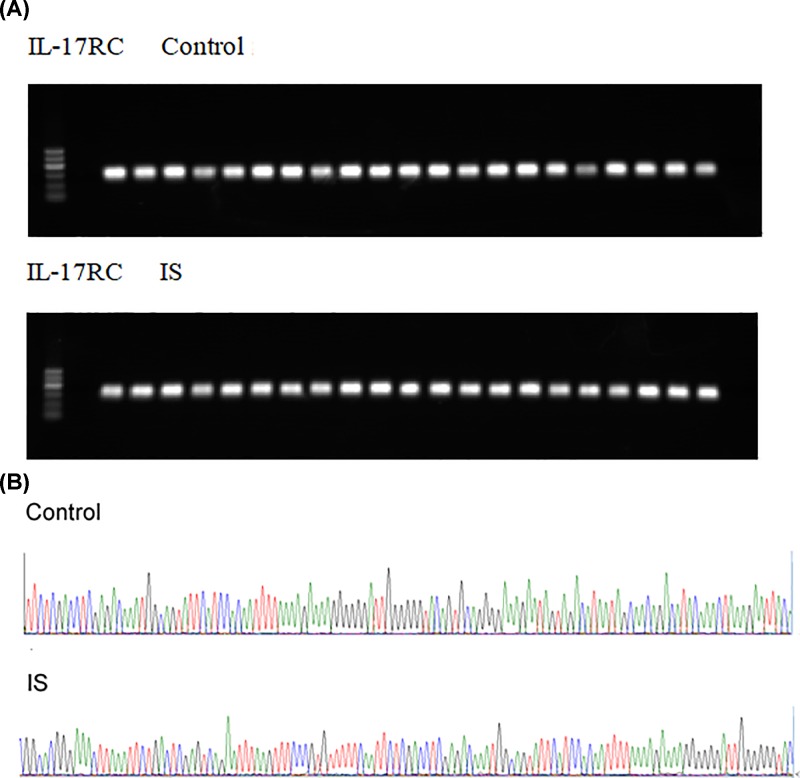
Identification of IL-17 RC genotype (**A**) PCR amplification of DNA samples from IS and control group. (**B**) Polymorphic sequencing of IL-17 RC genotype.

We designed IL-17RC PCR primer and PCR amplification was performed on the DNA of samples from IS group and the control group. Results showed the IL-17RC genotype was rs376511 (Figure 3A). Results of polymorphic sequencing are shown in Figure 3B.

### Comparison of IL-17RC genotype between IS and control

**Figure 4:**
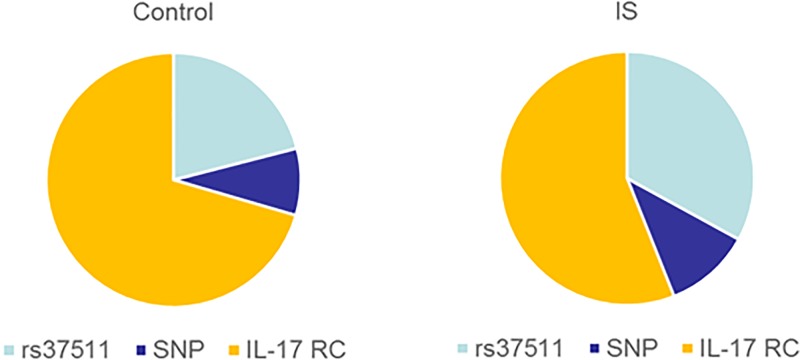
The proportion of IL-17RC, re37511 and SNP in the control group and IS group Proportion of re37511 in IS group was markedly higher than the control group.

To study of the relationship between rs376511 polymorphism and IS, we counted and compared the proportion of IL-17RC, SNP and rs376511 in IS and control group. Results were shown in Figure 4. As shown, the proportions of IL-17RC, SNP and rs376511 in IS group were 55.3, 7.3 and 37.4%, respectively. However, the proportions of IL-17RC, SNP and rs376511 in the control group were 72.4, 7.2 and 20.4%, respectively. The proportion of IL-17RC in the control group was significantly higher than IS group, while proportion of rs376511 in IS group was markedly higher than the control group. Therefore, we speculated that IL-17RC rs376511 was related with the occurrence and development of IS.

### Relationship between IL-17RC genotype and therapeutic effect and prognosis

**Figure 5:**
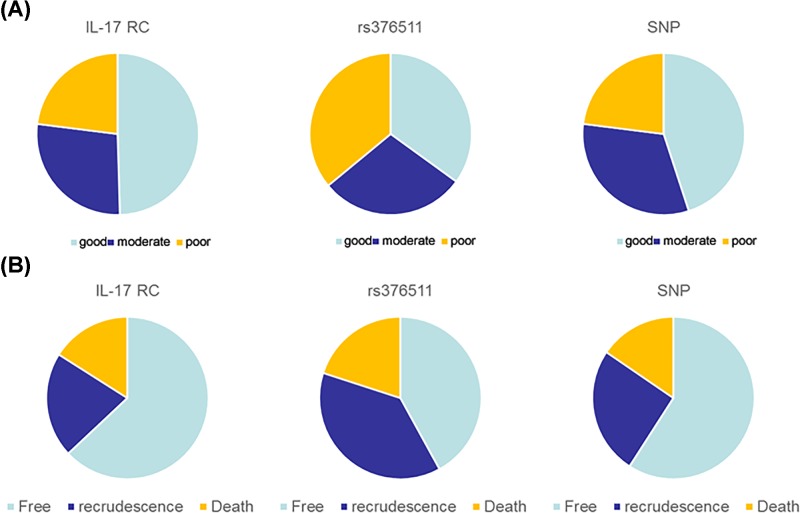
IL-17 RC genotype influences the treatment effect on IS and prognosis of IS (**A**) IL-17 RC re37511 reduces the treatment effect of IS. (**B**) IL-17 RC re37511 promotes and recrudescence and death of IS patients.

The relationship between IL-17RC genotype and therapeutic effect is shown in Figure 5A. As shown, the proportions of well, moderate and poor treatment in patients with IL-17RC were 50.3, 28.4 and 21.3%, respectively; the proportions in patients with rs376511 were 39.2, 40 and 20.8%, respectively; the proportions in patients with SNP were 46.3, 35.1 and 18.6%, respectively. From those results, we concluded treatment on patients with IL-17RC was well, while treatment on rs376511 was poor. The treatment effect on patients with SNP was between the effect of IL-17RC and SNP.

We estimated the prognosis of patients with different of IL-17RC genotype by following up patients for 3 years. Results are shown in Figure 5B. As shown, the percent of death in IL-17RC, rs376511 and SNP was 17.2, 22.5 and 18.8%, respectively; the percent of recrudescence was 18.1, 37.3 and 17.2%, respectively; the percent of free in IL-17RC, rs376511 and SNP was 64.7, 40.2 and 64.0%, respectively. Therefore, we speculated that patients with IL-17RC rs376511 polymorphism have a poor prognosis.

## Discussion

Stroke is an important reason for human disability, suffering and death. A lot of studies showed acute local inflammation is the characteristic of ischemic damage [8]. Highly expressed inflammatory markers can affect the development of ischemic vascular disease. In the occurrence and development of atherosclerosis, inflammation plays an important role [9]. Atherosclerosis often leads to IS because of the inflammation of blood vessels. So pro-inflammatory genes are quite important in the development of IS [10]. IL-17 consists of 155 amino acids and its molecular weight is ∼20 kDa. IL-17 can regulate inflammatory responses in many tissues. It can induce inflammation-related genes, such as IL-6 and leukemia inhibitory factor. IL-17 receptor family contains IL-17RA, IL-17RB, IL-17RC, IL-17RD and IL-17RE. IL-17 can play a physiological role only after binding to the receptor, while IL-17R can be expressed in endothelial cells. IL-17 can combine with IL-17R to activate NF-κβ and P-38, which then enhance the secretion of IL-6 and IL-8. Other studies have shown that IL-17 alone or combination with IL-1 and TNF-α can induce the synthesis of IL-6 and IL-8 in fibroblast-like synovial cells. IL-17C often acts on epithelial cells by secretion to enhance inflammatory cytokines levels, antimicrobial peptides and anti-apoptotic factors [11]. Studies have shown that respiratory epithelial cells can secrete IL-17C and promote the expression of human beta-defensin 2 (hBD2), CSF and S100A12 by autocrine and paracrine modes [6,12]. IL-17 can combined with its receptor to promote the formation of atherosclerotic plaques, while atherosclerosis is closely related with the occurrence of IS [13]. In our study, we used qRT-PCR to determine the relative mRNA levels of IL-17, IL-17RC, IL-6, IL-8, G-CSF and GM-CSF in serum samples from IS patients and the control healthy. Results showed the relative mRNA levels of IL-17, IL-17RC, IL-6, IL-8, G-CSF and GM-CSF in IS samples were significantly higher than those in the control, which indicated IS up-regulates IL-17 expression, which combined with IL-17RC to secret IL-6, IL-8, G-CSF and GM-CSF. Western blotting further verified the increased protein expression of IL-17 and IL-17RC in IS. Therefore, we speculated IL-17RC gene polymorphism may be related with the development of IS. Results of PCR amplification showed IL-17RC rs376511 was evident in IS group compared with the control. The percent of rs376511 in IS patients was markedly higher than that in the control group, while IL-17RC in IS patients was less. So we speculated IL-17RC rs376511 may influence the treatment effect and prognosis of IS. Our research showed treatment on patients with IL-17RC was well, while treatment on rs376511 was poor. The treatment effect on patients with SNP was between the effect of IL-17RC and SNP. After follow-up visits for 3 years, we obtained the death percent of patients with rs376511 which was much higher than IL-17RC and SNP, and the recrudescence percent of patients with rs376511 was 37.3%, which was higher than that with IL-17RC (18.1%) and SNP (17.2%).

In conclusion, IL-17 was up-regulated in IS, which combined with IL-17RC to secret IL-6, IL-8, G-CSF and GM-CSF. IL-17RC rs376511 polymorphism was closely related to the treatment effect on IS and prognosis of IS. Patients with IL-17RC rs376511 have a poor treatment and prognosis.

## Abbreviations

G-CSF: granulocyte colony-stimulating factor
GM-CSF: granulocyte-macrophage colony stimulating factor
hBD2: human beta-defensin2
IL-17R: interleukin-17 receptor
IL-17RA: interleukin-17 receptor A
IL-17RC: interleukin-17 receptor C
IS: ischemic stroke
qRT-PCR: Quantitative real time PCR
RFLP: restriction fragment length polymorphism
SNP: single nucleotide polymorphism
