# Correction: Interleukin-17 receptor C gene polymorphism reduces treatment effect and promotes poor prognosis of ischemic stroke

**DOI:** 10.1042/BSR-20190435_COR

**Published:** 2020-01-14

The authors of the published article “Interleukin-17 receptor C gene polymorphism reduces treatment effect and promotes poor prognosis of ischemic stroke” (*Biosci. Rep.* 39(10); DOI: 10.1042/BSR20190435) would like to correct the source of their patient cases, as stated in their Materials and methods. The correct study population source is presented below:

Between April 2015 and May 2016, 300 patients, who were recently diagnosed with ischemic stroke (IS), were recruited from The first affiliated hospital of Henan University of Science and Technology. Moreover, 300 healthy people were taken as control group. Both IS patients and the controls, gender and age-matched (±5 years) were selected. Before our study, all participants signed written informed consent. Our study protocol was also approved via the ethics committee of The first affiliated hospital of Henan University of Science and Technology. The serum of IS patients and controls were collected.

